# Role of the ubiquitin proteasome system in renal cell carcinoma

**DOI:** 10.1186/1471-2091-8-S1-S4

**Published:** 2007-11-22

**Authors:** Paul G Corn

**Affiliations:** 1 MD Anderson Cancer Center, GU Medical Oncology, Box 1374, 1515 Holcombe Boulevard, Houston, TX 77030, USA

## Abstract

Renal cell carcinoma (RCC) accounts for approximately 2.6% of all cancers in the United States. While early stage disease is curable by surgery, the median survival of metastatic disease is only 13 months. In the last decade, there has been considerable progress in understanding the genetics of RCC. The *VHL* tumor suppressor gene is inactivated in the majority of RCC cases. The VHL protein (pVHL) acts as an E3 ligase that targets HIF-1, the hypoxia inducible transcription factor, for degradation by the ubiquitin proteasome system (UPS). In RCC cases with mutant pVHL, HIF-1 is stabilized and aberrantly expressed in normoxia, leading to the activation of pro-survival genes such as vascular endothelial growth factor (VEGF). This review will focus on the defect in the UPS that underlies RCC and describe the development of novel therapies that target the UPS.

**Publication history:** Republished from Current BioData's Targeted Proteins database (TPdb; ).

## Role of the ubiquitin proteasome pathway in renal cancer

Each year in the United States, there are approximately 36,000 new cases of renal cell carcinoma (RCC) and 13,000 related deaths (statistics available at ) [1]. Though there are different pathologic subtypes, the majority (~75%) of RCC cases are referred to as “conventional” or “clear cell” type (CCRCC) [1]. Greater than 95% of clear cell kidney cancers occur sporadically within the population, while the remainder occur as part of relatively rare, inherited genetic syndromes including von Hippel-Lindau disease and familial clear cell renal cancer [1,2]. The primary genetic defect of clear cell kidney cancer (in both sporadic and hereditary forms) involves inactivation of the *VHL* gene pathway. Individuals with *VHL* disease harbor a germline mutation in one allele of the *VHL* gene and somatic inactivation of the remaining wild-type allele results in tumor development [3]. In sporadic CCRCC, somatic inactivation of the *VHL* gene also occurs in greater than 60% of cases via mutation, deletion or methylation-associated silencing [3-9]. *VHL* thus represents a classic tumor suppressor gene that is inactivated in CCRCC according to Knudsen's “two-hit” hypothesis [10,11]. Indeed, loss of *VHL* occurs at a very early stage in kidney cancer progression, suggesting that *VHL* represents the “gatekeeper” gene in this malignancy [12].

For decades preceding the modern era of genetics, surgeons and pathologists had described the richly vascular nature of RCC. When the *VHL* gene was originally identified in 1993, however, its function was not easily deduced from its structure because the amino acid sequence of the protein (pVHL) did not share any significant homology to other known proteins at the time [13]. It was subsequently discovered, however, that pVHL negatively regulates hypoxia-inducible genes such as vascular endothelial growth factor (VEGF) and erythropoietin (EPO) in renal cancer cell lines *in vitro*[14,15]. Normally, hypoxia-inducible genes such as VEGF are expressed at low or undetectable levels under normoxic conditions but are markedly induced under hypoxic conditions. In pVHL-deficient renal cancers, there is constitutive upregulation of hypoxic genes (including VEGF, erythopoietin and carbonic anhydrases) in normoxia [16-19]. Over the next decade, detailed biochemical, structural and functional analyses of pVHL identified its essential role as part of a multiprotein E3 ubiquitin ligase that targets specific proteins for destruction via the ubiquitin proteasome system (UPS) [3,15,20-23].

The UPS functions within normal cells of higher eukaryotes in two major ways: 1) as part of a degradative pathway that regulates the intracellular breakdown of proteins and 2) as part of a non-degradative pathway that regulates the location and activity of diverse cellular proteins [24-27]. The UPS is an integral part of normal cellular functions including cell cycle progression, signal transduction, response to extracellular stress and DNA repair (reviewed in 26) [26]. In addition, proteins that could be harmful to the cell, such as damaged, misfolded or misassembled proteins are also degraded [28].

**Figure 1:**
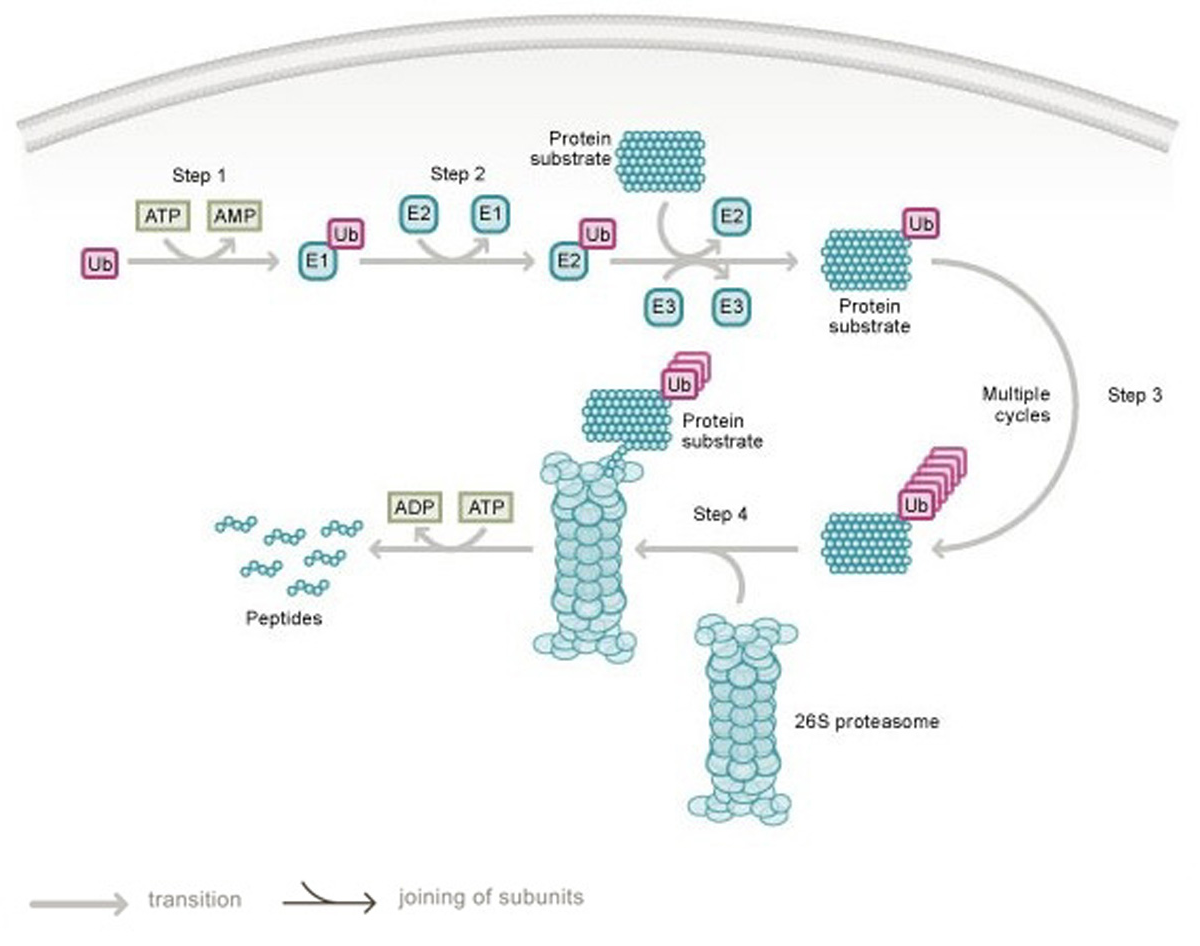
**The ubiquitin proteasome system**. In step 1, ubiquitin is activated by a ubiquitin activating enzyme, E1. In step 2, activated ubiquitin is transferred to a ubiquitin conjugating enzyme, E2. In step 3, ubiquitin is subsequently conjugated to target proteins in a process mediated by an E3 ubiquitin ligase. In step 4, the polyubiquitylated substrate protein is degraded by the 26S proteasome. A single E1 enzyme can transfer ubiquitin to all of the E2s in the cell, and each of the E2s associates with a restricted set of E3s that confer substrate specificity.

The mechanism for proteolysis by the UPS is highly regulated and involves several steps that depend on ubiquitin, a 76 amino acid protein that is highly conserved among higher eukaryotes [29]. In the first step, the proteolysis pathway is initiated by a ubiquitin activating enzyme (E1) that uses ATP to form a high-energy thiolester bond with the C-terminus of ubiquitin [30]. In the second step, activated ubiquitin is transferred to a ubiquitin conjugating enzyme (E2) [31]. In the third step, ubiquitin is subsequently conjugated to target proteins in a process mediated by an E3 ubiquitin ligase [32]. The E3 ligase serves as an adaptor molecule that interacts with both the target protein and the E2, resulting in formation of an isopeptide bond between the C-terminus of ubiquitin and an ε-amino group of lysine residues in the target protein. Successive transfers of activated ubiquitin to lysine-48 of the previously conjugated ubiquitin molecule lead to the formation of polyubiquitin chains [33,34], which serve as recognition markers for the 26S proteasome [35] (Figure 1).

Conversely, while the UPS degradative pathway involves polyubiquitylation of target proteins for degradation, the non-degradative UPS pathway generally involves monoubiquitylation of proteins [36,37]. Proteins can be modified on a single lysine with a single ubiquitin moiety or modified on multiple lysines with a single ubiquitin moiety (multi-monoubiquitylation). Examples of monoubiquitylation function include translocation of monoubiquitylated p53 to the mitochondria, endocytosis of monoubiquitylated receptor tyrosine kinases and activation of monoubiquitylated transcription factors [38-40].

The 26S proteasome is a ~2.5 MDa complex composed of a single core catalytic 20S particle capped on each end by a 19S particle [24]. The 20S particle is a barrel shaped structure comprised of four stacked rings, two outer α rings and two identical inner β rings. Each α and β ring is in turn composed of seven distinct subunits. The 20S particle has proteolytic sites facing the interior chamber of the barrel that are accessible via a narrow pore opening at either side [41]. Folded proteins, however, cannot access these pores, so native proteins have to be processed prior to degradation. Processing occurs via the 19S particle, which is comprised of at least 17 proteins ranging in molecular weight from 25 to 100 kDa. The 19S particle provides multiple functions necessary for binding, unfolding and processing of protein substrates prior to entry and degradation within the 20S particle [42,43]. In addition, the 19S particle contains six different ATPases that harvest the energy required for proteolysis [44].

The eukaryotic 20S particle contains chymotrypsin-, trypsin-, and caspase-like proteolytic enzyme activity [45-47]. Studies utilizing specific chemical inhibitors suggest that the chymotrypsin-like activity is the most important of the three enzyme activities for proteolysis [48]. In the final step of proteolysis, polyubiquitylated target proteins bind to the 19S particle where they are deubiquitylated, unfolded, delivered into the catalytic core of the 20S particle and degraded to peptides 3–22 amino acids in size [49,50]. The ubiquitin moieties are recycled for subsequent reactions.

Interestingly, the ubiquitin system is hierarchical: a single E1 enzyme can activate a larger number of E2s and each E2 can interact with one to several E3 proteins that ultimately tag the protein for degradation [51,52]. Some E3 ligases are single polypeptides, while others exist as multi-protein complexes. Although the absolute number of E3 ligases in mammalian cells remains to be determined, at least several hundred E3 ligases at the end of this enzymatic cascade are thought to confer specificity to the UPS [52].

Using biochemical methods, a link between pVHL and the cellular ubiquitylation machinery was made, supported by the discovery that pVHL exists in a complex with Elongin B, Elongin C, Cul2 and Rbx1 [53-55]. This complex closely resembles SCF complexes in yeast that contain homologues including Skp1 (Elongin C), Cdc53 (Cul2), and Roc1 (Rbx1). In yeast, the SCF complex targets proteins for ubiquitin-mediated proteasomal degradation and the F-box component (Roc1) confers substrate specificity. Subsequent functional studies supported the notion that the pVHL–Elongin B–Elongin C–Cul-2–Rbx1 complex functions as an E3 ligase [20-22]. While there is little sequence homology between pVHL and an F-box protein, both have an overall pattern of hydrophobic amino acids that appears to be important for target substrate recognition [56]. These seminal observations subsequently led to the important discovery that pVHL binds to HIFα proteins and targets them for ubiquitin-mediated proteolysis [15].

In hypoxia, one of three HIFα proteins (Hif1α, Hif2α or Hif3α) associates with Hif1β to form a heterodimeric transcription factor (HIF-1) that binds to a consensus hypoxia-responsive element (HRE; 5'-RCTGTG-3') and activates a number of (greater than 60) different target genes involved in diverse biologic processes including angiogenesis, cell cycle control, proliferation and energy metabolism (reviewed in 57) [57]. While Hif1β is constitutively expressed, HIFα levels are tightly regulated in response to changes in oxygen tension. Under hypoxic conditions, pVHL does not bind to HIFα proteins, which then accumulate and bind to Hif1β to activate transcription. When cells are shifted to normoxia, however, HIFα proteins instantaneously undergo a post-translational modification resulting in hydroxylation of crucial proline residues within regions of HIFα called oxygen-dependent degradation domains (ODDs) [23,58,59]. Hydroxylation results in a change in conformation of the HIFα proteins, which permits binding to and degradation by pVHL. Interestingly, hydroxylation of HIFα proteins is performed by a conserved family of prolyl-4 hydroxylases that require oxygen for activity, suggesting that these enzymes contribute to oxygen sensing [60,61].

**Figure 2:**
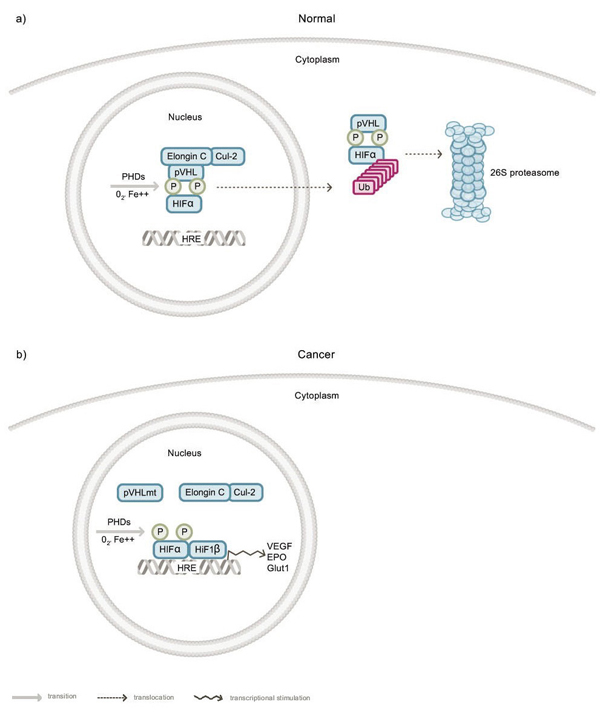
**Model for the E3 ligase function of pVHL in normoxia**. *A* In normal cells, HIFα proteins are hydroxylated by prolyl-4 hydroxylases (PHDs) that require oxygen for activity. pVHL, in a complex with multiple proteins including Elongin C and Cul-2, binds to hydroxylated HIFα proteins and delivers them to the 26S proteasome for destruction. *B* In RCC, *VHL* gene mutations often disrupt pVHL–HIFα binding and/or the pVHL–Elongin C–Cul-2 complex. The consequence is that stable HIFα proteins dimerize with Hif1β and the resulting HIF-1 complex binds to a hypoxia-response element (HRE) to activate pro-survival genes, such as VEGF, EPO and Glut1.

X-ray crystallographic analysis of pVHL has revealed two major protein domains: an α domain and a β domain. The surface of the α domain (residues 155–192) is primarily responsible for the interaction between pVHL and Elongin C [56]. The surface of the β domain consists of a seven-stranded β sandwich (residues 63–154) and an α helix (residues 193–204), and is primarily responsible for binding to HIFα proteins. In sporadic RCC, about one half of *VHL* gene mutations map to the α domain and the other half to the β domain. The majority of these mutations () are missense mutations and many lead to aberrant upregulation of HIF-1, either by abolishing binding of pVHL to Elongin C and/or to HIFα proteins (reviewed in 3) [3,62,63]. In patients with inherited VHL disease, RCC tumors harbor *VHL* deletions or truncation mutations, also leading to aberrant upregulation of HIF-1. Taken together, these observations support a genotype-phenotype link in RCC, since the hypervascularity of these tumors can be explained by a pVHL-dependent defect in ubiquitin-mediated degradation of HIFα proteins, leading to increased HIF-1 transcriptional activity with consequent upregulation of VEGF and other factors that are thought to promote survival (reviewed in 57) [57,64-66] (Figure 2).

## Models for studying RCC

Most of the data described above linking pVHL function to the UPS was obtained from studies conducted *in vitro*. Evidence for this model evolved and converged over time from a variety of disciplines including biochemistry, structural biology and molecular genetics. Invaluable insights were gained from studies of yeast, *Drosophila, Caenorhabditis elegans* and human RCC cell lines [20,54,67-69]. Our understanding of the genotype-phenotype link in RCC is based on a thorough analysis of *VHL* mutations found in primary human kidney tumors [63].

Historically, there has been considerable difficulty in establishing relevant animal models for RCC. *VHL*-/- mice (created by the Linehan laboratory, National Cancer Institute, USA) die during early embryogenesis due to defective placental vasculogenesis [70]. *VHL*+/- heterozygous mice (created by the Walker laboratory, University of Texas M.D. Anderson Cancer Center, USA) are prone to vascular proliferative lesions of the liver but do not develop kidney tumors [71]. Rodents exposed to carcinogens can develop kidney tumors; however, the genetic defect involves the tumor suppressor gene *Tsc-2* (tuberous sclerosis complex-2) rather than *VHL* and the animals display chromophilic rather than clear cell histology [71]. Interestingly, though, *Tsc-2* mutant tumors, like *VHL* mutant tumors, are highly vascular and express very high levels of HIF-1 [72].

Several studies have generated mice with human RCC cell line-derived xenograft tumors, injected either subcutaneously or orthotopically into the kidney [69,73-75]. While animal models have a limited capacity to recapitulate the tumor biology of humans, they have permitted controlled experiments both for the analysis of renal tumorigenesis and the evaluation of novel therapeutics for RCC. For example, when pVHL function is restored to renal cancer cell lines lacking pVHL, these cells lose the capacity to form tumors in mice *in vivo*[69]. However, when these same pVHL-restored RCC cells are engineered to express a stable Hif2α variant lacking its prolyl hydroxylation/pVHL binding sites, they regain their ability to form tumors *in vivo*[76]. These data underscore the importance of HIF-1 signaling in *VHL*-derived tumorigenesis.

## Disease targets and ligands

The Food and Drug Administration recently approved two new agents for the treatment of advanced kidney cancer: Sutent (Sunitinib, SU011248, manufactured by Pfizer) and Nexavar (Sorafenib, Bay 43-9006, manufactured by Bayer Pharmaceuticals and Onyx). Both agents are small molecule tyrosine kinase inhibitors that block receptor signaling by primarily targeting VEGF and platelet-derived growth factor (PDGF) [77]. It appears, however, that other tyrosine kinases are targeted, for example c-Kit, which is inhibited by Sutent [78]. The aggregate effect of these drugs is to inhibit angiogenesis, although direct effects on cell proliferation may also be important. Along with Avastin (Bevacizumab, manufactured by Genentech), the anti-VEGF antibody, these agents demonstrate the therapeutic benefit of inhibiting angiogenesis in RCC [79].

The discovery that the primary genetic event in CCRCC (loss of *VHL*) results in a defect in the UPS suggests that novel therapies targeting this pathway could be employed to induce apoptosis in cancer cells. Importantly, transformed cells generally display increased susceptibility to apoptosis by proteasome inhibitors when compared with non-transformed cells [80]. The basis for this is under investigation, but possible explanations include stabilization of proteins that normally either contribute to apoptosis (for example p53, p21, p27, Bax and Smac/Diablo) or that antagonize pro-survival pathways (for example Iκβ, proteasomal stabilization of which inhibits nuclear translocation of NFκβ, a proto-oncogenic transcription factor) [81].

The discovery of drugs that inhibit the UPS is proceeding rapidly. To consider their potential use in RCC, it is helpful to discuss them based on the component of the UPS they target.

### Inhibitors of the 26S proteasome

Bortezomib (Velcade, manufactured by Millennium Pharmaceuticals) is a dipetidyl boronic acid that reversibly inhibits the chymotryspin-like activity found within the 20S particle of the proteasome [82]. Bortezomib has already been established as an effective agent in the treatment of multiple myeloma [83]. The drug causes apoptosis of kidney cancer cell lines *in vitro*, but was less promising in two Phase II clinical trials [84-86]. A study from Memorial Sloan-Kettering reported four partial responses from a total group of 37 RCC patients treated (25 clear cell, six papillary, one collecting duct and one medullary), with clear cell histology present in three of the four responders [85]. A study from the University of Chicago reported one partial response in a patient with clear cell histology from a total group of 21 RCC patients treated (histologic subtypes were not fully reported) [86]. Toxicities attributed to bortezomib (including fatigue, sensory neuropathy, nausea, anemia and transaminitis) probably reflect the consequences of nonspecific inhibition of the UPS. While neither study supported the use of bortezomib as a single therapeutic agent in patients with metastatic kidney cancer, the possibility of combining it with other agents in the future could still be theoretically desirable, since proteasome inhibition can enhance chemotherapy and overcome drug resistance in preclinical models [80,87].

### Inhibitors of E3 ubiquitin ligases

As described above, E3 ubiquitin ligases are very specific in their interaction with protein substrates. Therefore, they are attractive targets for drug discovery since their inhibition would be expected to have fewer “off-target” effects and less toxicity than inhibitors of the 26S proteasome. The interaction between the p53 tumor suppressor gene and its E3 ligase, MDM2, provides an illustration of this concept. The p53 tumor suppressor pathway is inactivated in the majority of human tumors. Approximately 50% of all human tumors contain mutations in the p53 gene and the remainder (with wild-type p53) display perturbations in p53 signaling due to increased proteasomal degradation of p53 [88]. Increased proteasomal degradation of p53 occurs because of increased expression of MDM2, or alternatively, loss of Arf, an inhibitor of the p53–MDM2 interaction.

The Nutlins (cis-imidazoline derivatives, available from Sigma) are small molecule inhibitors of MDM2 that were discovered in a chemical library screen [89]. Structurally, the Nutlins occupy the p53 binding site of MDM2, thereby displacing p53 and preventing its destruction [90]. Increased levels of p53 then restore tumor suppressor function. The potential for Nutlins (which are orally bioavailable) as anti-cancer agents is now being explored in pre-clinical studies. Importantly, in tumor xenografts derived from a human osteosarcoma cell line, Nutlins suppressed tumor growth with minimal toxicity to normal tissues [91].

Development of small molecules that affect the pVHL–HIFα interaction is also a theoretic possibility. However, a conceptual dilemma immediately presents itself. In the case of p53 and MDM2, the UPS defect is inappropriately *increased* degradation of p53, with both proteins in their wild-type conformation. In the case of pVHL and HIFα, the UPS defect is inappropriately *decreased* degradation of HIFα due to mutant pVHL. In the case of p53–MDM2, the goal of a small molecule is to *inhibit* binding of p53 to MDM2. By contrast, in the case of pVHL–HIFα, the goal would be to *promote* binding of a mutant form of pVHL to HIFα. Drugs that promote, rather than inhibit, protein–protein interactions are extremely difficult to design and screen for. In addition, given the large number of *VHL* mutations that would presumably result in different conformations of pVHL, the development of small molecule agonists to promote a mutant pVHL–HIFα association would in reality be unfeasible.

### Activators of the 26S proteasome

Since loss of pVHL E3 ligase function leads to impaired ubiquitylation and protein degradation in RCC, the development of drugs that activate proteasome function would theoretically be of interest [92]. However, a potential limitation of this strategy would be the requirement that such drugs enhance degradation of non-ubiquitylated proteins. While such non-specific “panactivators” of proteasome function have not been described, drugs are being developed that can promote ubiquitin-mediated proteasomal degradation of HIFα subunits in pVHL-deficient RCC.

### Inhibitors of Hsp90

Hsp90 is described as a “super-chaperone machine” because it normally associates with certain proteins to promote their proper folding so that they can respond to a stimulus (e.g. cytosolic kinase) or bind to a ligand (e.g. a steroid hormone) [93]. Hsp90 also functions to stabilize certain proteins from proteasomal degradation. In some cases, Hsp90 stabilizes proteins that function as oncogenes in tumorigenesis, examples of which include the p210^Bcr-Abl^ protein in chronic myelogenous leukemia, HER-2 in breast and prostate cancer, and Hif1α [94-98]. Importantly, small molecule inhibitors of Hsp90 promote degradation of HIFα proteins in a pVHL-independent manner [99,100]. Interestingly, this process involves ubiquitylation of HIFα subunits via a mechanism that does not involve proline hydroxylation [99]. Several Phase I and/or Phase II studies are being conducted in the United States testing the Hsp90 inhibitor 17-DMAG (17-N-Allylamino-17-Demethoxygeldanamycin, available from A.G. Scientific) in patients with RCC (see ).

### Inhibitors of mTOR

The mTOR (mammalian target of rapamycin) signaling pathway has been shown to enhance HIF-1 activity in response to growth factors [101,102]. Inhibitors of the mTOR pathway, such as rapamycin, reduce HIF-1 levels and HIF-1 transcriptional activity. The mechanism of the suppressive effect of rapamycin on HIF-1 activity is due to increased degradation of HIFα proteins by the UPS [103]. Early clinical trials have shown that the novel mTOR inhibitor CCI779 (temsirolimus, Torisel, manufactured by Wyeth Pharmaceuticals), has promising activity in patients with advanced RCC [104,105].

## Future directions in the treatment of RCC

Continued efforts to identify drugs that activate the proteasome pathway rather than inhibit it would address the fact that the primary UPS defect in RCC is its downregulation rather than upregulation. Upregulation of the UPS system has been reported in other cancer types and has been offered as one reason why these cancers are more susceptible than normal cells to apoptosis induced by proteasome inhibitors [80,87]. While drugs that specifically target HIF-1 degradation exist, there have been no reports to date of compounds that generally increase protein turnover by the UPS. Since the pVHL E3 ligase complex targets proteins other than HIFα, for example atypical protein C, such compounds would be an interesting concept [106].

A major obstacle in treating advanced RCC tumors (i.e. those not cured by surgery) has been their resistance to most standard chemo- and radio- therapies. The reasons for this are unclear, though possibilities include the complex genetics of the tumor, defective angiogenesis and tumor hypoxia. Even Sutent and Nexavar, which have upstaged low-dose immunotherapy in the treatment of this disease, are predominantly tumoristatic rather than tumoricidal. Thus, there is considerable room for improvement in treating RCC. One prediction is that combinations of two or more drugs will likely be necessary to approach any possibility of a cure. As an example, it makes biologic sense that the combination of a drug that blocks VEGF receptor signaling, an Hsp90 inhibitor and a DNA damaging agent could synergistically promote apoptosis in RCC. However, the exact combination of drugs and the ability to administer them safely to human patients remains a daunting challenge.

## List of abbreviations

UPS = ubiquitin proteasome system, VHL = von Hippel-Lindau, RCC = renal cell carcinoma, CCRCC = clear cell renal cell carcinoma, ODD = oxygen-dependent degradation domain

## Competing interests

The authors declare that they have no competing interests.

## Publication history

Republished from Current BioData's Targeted Proteins database (TPdb; ).
